# Extracellular volume fraction is associated with B-type natriuretic peptide in hypertrophic cardiomyopathy

**DOI:** 10.1186/1532-429X-16-S1-P331

**Published:** 2014-01-16

**Authors:** Timothy C Wong, Jeffrey Chung, Peter Kellman, Erik B Schelbert

**Affiliations:** 1 Department of Medicine, Division of Cardiology, University of Pittsburgh School of Medicine, Pittsburgh, Pennsylvania, USA; 2 Cardiovascular Magnetic Resonance Center, UPMC, Pittsburgh, Pennsylvania, USA; 3 Hypertrophic Cardiomyopathy Center, UPMC, Pittsburgh, Pennsylvania, USA; 4 Laboratory of Cardiac Energetics, National Heart Lung and Blood Institute, NIH, Bethesda, Maryland, USA

## Background

Hypertrophic cardiomyopathy is a common cardiovascular genetic disease characterized by sarcomeric gene mutations which lead to findings of cardiac hypertrophy, myocyte disarray, and fibrosis. While late gadolinium enhancement (LGE) cardiovascular magnetic resonance (CMR) detects focal, macroscopic regions of replacement fibrosis non-invasively, novel T1 CMR measurement techniques including extracellular volume fraction (ECV) diffuse interstitial fibrosis throughout the myocardium. Plasma B-type natriuretic peptide levels are often elevated in situations of increased wall tension and volume overload. Given that such states may be associated with myocardial fibrosis, and because BNP levels provide independent prognostic insight in HCM, we sought to determine the association between BNP and ECV measurement by CMR.

## Methods

We recruited 50 consecutive patients referred to the UPMC Hypertrophic Cardiomyopathy Center and UPMC Cardiovascular Magnetic Resonance Center for clinical evaluation to participate in a prospective cohort formed to describe the association between CMR data and outcomes. Contemporaneous echocardiography, treadmill stress echocardiography, and clinical evaluation data were recorded. BNP levels were obtained as part of routine clinical care or drawn the same day as CMR study using research funding. We computed ECV from measures of pre and post contrast T1 of mid-myocardium and blood (short axis prescriptions at the base and mid ventricle) using modified Look-Locker inversion recovery (MOLLI) pulse sequences. BNP levels were natural log transformed given their skewed distribution. Univariable and multivariable regression models tested for associations between markers of cardiac remodeling as well as other predictors of BNP (including age, left ventricular mass(LVM), left ventricular outflow obstruction, body mass index(BMI), findings of late gadolinium enhancement, ECV). Models were constrained to 4 independent variables to avoid overfitting.

## Results

**Figure 1:**
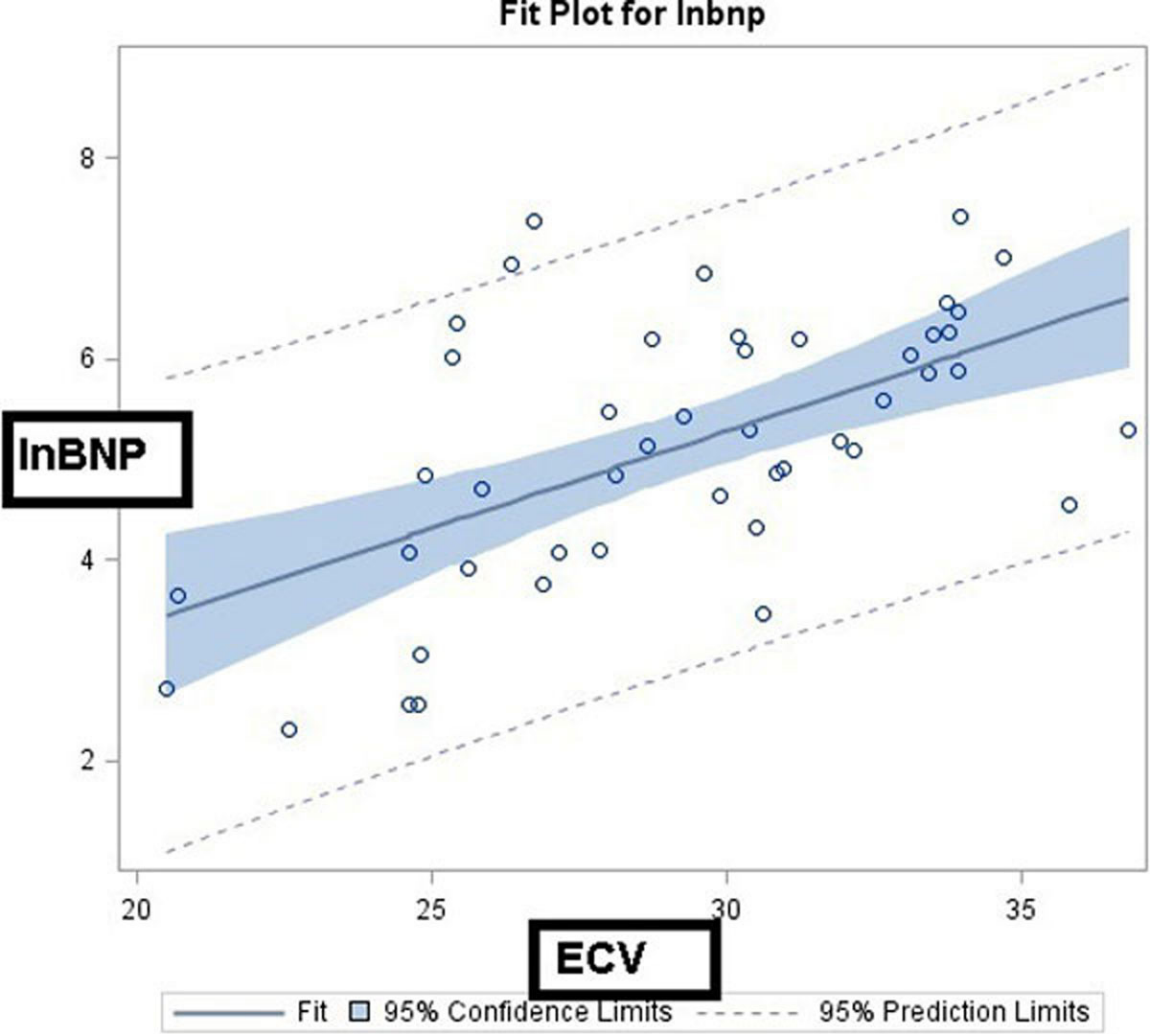


There was a moderate correlation (r = 0.58, p < 0.0001) between lnBNP and ECV (Figure 1). ECV remained significantly associated with lnBNP after adjusting for LGE, LVOT obstruction, BMI, and LVM. When analyses were stratified by the presence or absence of LGE, ECV remained significantly associated with lnBNP (Table 1).

**Table 1:** Associations with lnBNP by LGE status

	LGE absent			LGE present		
	**β**	**t**	**p value**	**β**	**t**	**p value**

ECV (%)	0.33	5.9	< 0.001	0.11	2.24	0.033

LVOT obstruction (> 35 mm Hg)	0.02	0.06	0.951	-0.05	-0.14	0.894

LV mass (g)	0.01	4.24	0.002	0.01	2.17	0.038

## Conclusions

We found a novel association between lnBNP and ECV by CMR, after adjustment for confounding variables. The relationship persisted even after including LGE in regression models and stratifying by its presence. BNP and ECV are both strong predictors of adverse events. Detection of diffuse interstitial fibrosis by ECV may be a meaningful imaging biomarker to characterize HCM status, progression, and outcomes and warrants further study.

## Funding

American Heart Association, Pittsburgh Foundation.

